# Quality, Microstructure and Properties of Metal Alloys (Second Volume)

**DOI:** 10.3390/ma18225085

**Published:** 2025-11-09

**Authors:** Tomasz Lipiński

**Affiliations:** Department of Materials and Machine Technology, Faculty of Technical Sciences, University of Warmia and Mazury in Olsztyn, 10-719 Olsztyn, Poland; tomaszlipinski.tl@gmail.com

The quality, properties, and related application possibilities of structural materials result directly from their microstructure, which, in turn, is shaped by the selected chemical composition of metal alloys and manufacturing processes. Over the course of several centuries, humans have explored available natural materials, subsequently producing materials for engineering. Over time, available natural materials have become insufficient to meet our ever-growing needs. The development of a group of engineering materials now called classical materials has enabled the creation of machines and devices. The ongoing advancement of civilization has both fostered developments and necessitated further research aimed at expanding scientific understanding and, consequently, enriching the development of structural materials. New groups of materials with differing properties have begun to emerge. The development of research methods combined with new material production technologies has enabled new, more favorable functional properties to arise in already known materials. Further improvements in research and manufacturing processes have enabled the control of material microstructures, and by appropriately shaping these, it has been possible to obtain materials with properties specific to certain applications.

The materials available from manufacturers come with a very wide range of qualities. These include not only the broadly understood properties of these materials but also the economic and ecological factors of their production, use, and subsequent recycling. Designers, selecting materials for specific needs, place increasingly stringent requirements on them. This necessitates the continuous improvement of existing materials while simultaneously developing new ones. Experience and available knowledge about the behavior of individual materials play a significant role in the material selection process. For these reasons, introducing a completely new material to the market is not easy. It is necessary not only to develop a new material but also to present reliable research results confirming its suitability for use and its operational reliability. Unfortunately, even high-quality materials, products, or products manufactured using advanced technology are not always accepted by the market. Despite their excellent performance characteristics, the popularity of materials and products made from them often depends not only on price and appearance but also on the local preferences and tastes of the target market. Market requirements and preferences must be continuously monitored and analyzed. Conclusions drawn from market demand and demand analysis primarily determine the quantity and quality of production. This is, of course, carried out with respect to the technical and economic possibilities of production.

There are many known applications of metal alloys. These alloys have been divided into groups. The basic groups of metal alloys include casting alloys and wrought alloys. Further possibilities for shaping the microstructures of these groups depend on their initial state, primarily their microstructure. By appropriately selecting their chemical composition, the microstructure of metal alloys can be shaped through appropriate heat treatment and a combination of several different types of treatment, such as thermochemical. By appropriately selecting the chemical composition and manufacturing processes, it is possible to obtain materials with single-phase or multi-phase crystalline microstructures, metallic glasses, etc. Complex operating conditions and economic factors require the production of materials with a surface layer containing different properties from the rest of the alloy. This is an increasingly common direction of research into improving structural materials. Dynamic technological development also enables the production of materials using modern technologies, such as additive manufacturing.

Engineering materials are manufactured from resources available in the Earth’s crust. Their production is based on needs, research, experiments, and knowledge analysis, but also on trial and error. At the current level of technological development, available computer programs are also used to predict material properties. However, shaping the functional properties of structural materials is still based on their chemical composition and the conditions under which they are manufactured during technological processes. Improving the chemical composition of known metal alloys, considering the new scientific achievements involving the development of new compounds in terms of their chemical composition, granulation, and form, and improving the manufacturing process to shape their microstructures, facilitates the introduction of newer and more advanced materials. Unfortunately, not all materials with favorable functional properties can be used on an industrial scale. This is due, to the availability of individual components and the complex processes of alloy and layer production, among other factors, but above all, economic factors are crucial in the widespread use of a given material. These factors necessitate the optimization of the chemical composition as well as material manufacturing processes. This can be achieved by combining several forms of insight, including experience and knowledge gained from fundamental research, materials science, and materials engineering. The existing research framework is still far from perfect. Consequently, some relationships affecting material properties are difficult to clearly delineate. Therefore, it is necessary for researchers to conduct studies that allow for the formulation of hypotheses and that meaningfully identify areas for further research.

The brief considerations presented above demonstrate that the properties of a metallic structural material are shaped by the microstructure of the metal alloy used; these metal alloys are obtained by capitalizing on the achievements made in various areas of knowledge. Therefore, it is crucial to link the quality of metal alloys with their microstructure and properties. Examples of research results in this area are presented in this second edition of ‘Quality, Microstructure and Properties of Metal Alloys’, which is expands of the first edition [1].

In [2], the authors attempted to deposit a high-entropy alloy (HEA) composed of 45% Ni, 20% Co, 10% Fe, 10% Cr, 7.5% Al, and 7.5% Ti on SUS 304 substrates using direct energy deposition (DED). They analyzed the quality of the resulting material based on its microstructure and mechanical properties. The addition of aluminum and titanium creates a γ + γ′ phase structure within a multicomponent FCC–HEA matrix, enhancing the thermal stability and coarsening the resistance and strength. The γ′ phase with an ordered FCC structure significantly improves the mechanical properties. Analysis confirmed the presence of the γ + γ′ structure and demonstrated the alloy’s high tensile strength and microhardness. The γ′ phase with the FCC network was identified as widely dispersed in the form of pore-like dots. The mechanical properties correlated with the microstructure and resulted in the following averages: Re = 514 MPa, Rm = 631 MPa, and hardness = 370 HV.

In [3], the authors noted that surface development had a significant effect on the adhesion and microstructure of the deposited coating, but a clear explanation of the effect of substrate surface preparation on the quality of the deposited coating remains lacking. They investigated the effect of various surface pretreatment methods used for Al 7075 alloy on the microstructure and mechanical properties of cold-sprayed nickel coatings. The authors demonstrated that the form of surface preparation affected the adhesion and microstructure of the coatings. They also demonstrated the effect of deposition temperature on the microstructure and properties of the layer. At a high gas temperature, a dense microstructure was obtained, demonstrating the very good adhesion of impacting powder particles to the substrate and good bonding between the deposited layers. They observed various shapes and a large variation in the sizes of Ni grains, ranging from several dozen to several hundred nanometers. The grain shape also oscillated from equiaxed to elongated. With increasing deposition temperatures, a significant increase in coating thicknesses was observed, this being a result of the higher velocity of the powder grains. At a higher gas temperature, an increase in the layer hardness was observed, both on the micro and macro scale, as was an increase in adhesion and the elastic modulus. The adhesion strength of the Ni coatings, determined in the study at temperatures ranging from 500 °C to 800 °C, increased with increasing substrate surface development. After the deposition of the Ni coatings on abrasive blast-treated Al 7075 substrate with the highest roughness at a gas temperature of 800 °C, the maximum adhesion achieved in the experiment was 44.6 MPa.

Analyzing the effect of the rare-earth element La on the microstructure and mechanical properties of the Al-5.4Cu-0.7Mg-0.6Ag silumin, it was found that the introduction of small amounts of La to the aluminum–silicon casting alloy significantly reduced the grain size [4]. This led to an improvement in the alloy’s mechanical properties. A concentration of 0.4 wt.% La was indicated, for which the alloy’s mechanical properties, represented by strength and elongation, both at room temperature and at temperatures elevated up to 350 °C, were higher than those of the alloy without the La addition. The addition of 0.4% La increased the tensile strength at ambient temperature from 380 to 411 MPa and the elongation from 5.4 to 7.9%, while at 350 °C, the tensile strength increased from 120 to 146 MPa, and the elongation decreased from 9.3 to 8.3%. At this temperature, a maximum elongation of 11.3 was observed for 0.6% La, at which the strength was 126 MPa. The authors found that the addition of La reduced the size of the Ω-strengthening precipitate phase while simultaneously increasing its precipitate density. A higher La content led to the appearance of the Al6Cu6La phase, which resulted in reduced Ω-phase precipitates. These microstructural changes resulted in changes in the mechanical properties of the treated alloy.

The authors of [5] developed a quantitative prediction model for Young’s modulus based on changes occurring in the microstructure of the 800H alloy. These observations revealed typical microstructural evolution processes due to creep. Based on their analysis, the authors predicted changes in the microstructure and properties of the steel, with an accuracy exceeding 95%. The forecast error is estimated at 1.59% when compared to the experimental data. According to the authors, the presented research results not only explain the relationships between the microstructure and macroscopic properties of the steel but also provide a quantitative and theoretical basis for the results obtained from destructive testing. The paper presents and explains the changes occurring in the microstructure using the example of alloy 800H and can also support the interpretation of non-destructive testing for the assessment of creep damage.

In [6], an attempt was made to analyze the properties of the IN718 alloy, which is highly sensitive to microstructural features. The microstructure of IN718, like any alloy, is largely shaped by the manufacturing process and subsequent heat treatment. The literature on the main microstructural features of IN718 alloy produced by Laser Powder Bed Fusion (LPBF) was analyzed. The creep mechanisms, microstructure description, and the most effective heat treatment methods for achieving it were analyzed. The analysis was conducted with a view to its effect on creep strength. The authors report that there is no universally accepted heat treatment method, and the microstructural features that optimize creep resistance are still a matter of debate. Based on the literature, the optimal creep parameters were determined to be as follows: a high density and an optimal size of γ′/γ″ strengthening precipitates; the dissolution of harmful Laves and δ phases; large equiaxed grains; the presence of twin boundaries and subregions, if possible; a lack of excessive carbide thickening; and low porosity. According to the authors, the above microstructural parameters can be achieved by homogenizing the alloy at a temperature above 1060 °C in combination with double-agging, or by hot isostatic pressing. For hot isostatic pressing methods, grain growth and reduced porosity are expected.

The results of research on the change in the microstructure of alloys as a result of recrystallization [7,8] and plastic deformation [9,10] were also presented. The development of the primary γ’ phase and the density of geometrically necessary dislocation (GND) was investigated via annealing tests in a deformed single-crystal third-generation nickel superalloy, WZ30, after 5% compressive strain, as a function of temperature [7]. It was shown that the effect of isochronous annealing (700–1200 °C/min) on the microstructure exhibited temperature-dependent changes. After annealing the alloy at 1000 °C, a non-monotonic decrease in the density of geometrically necessary dislocation was observed, while 62.7% of the main γ’ phase was retained. Extending the annealing time at 1000 °C for 10 h completely prevented recrystallization during subsequent dissolution at 1340 °C.

High-temperature deformation analysis of 5383 aluminum alloy with varying Mg compositions was undertaken in [8]. It was shown that a 4–5% Mg addition increases the peak flow stress of the 5383 aluminum alloy. This occurs due to increased crystal lattice deformation as well as dislocation blocking and second-phase interactions. Further increasing the Mg content reduces stress. The authors attribute this to enhanced dynamic softening mechanisms. Increased Mg content causes the expansion of the instability region, narrowing the safe processing windows from Mg 4.0 (390~500 °C/0.05~0.4 s^−1^) to Mg 5.0 (420~500 °C/0.05~0.16 s^−1^). This occurs due to the thickening of the iron-rich phase, which enhances local dislocation accumulation. The authors conclude that higher temperatures and lower strain rates are necessary to achieve stability.

An analysis of the orientation-dependent mechanical behavior of the BCC-Fe cell was also conducted, providing new insights into the thermokinetic synergy during deformation based on atomic configurations and stress–strain curves obtained from molecular dynamics MD simulations and its causes in light of the thermokinetic synergy for dislocation motion [9]. The authors concluded that the anisotropic mechanical behavior and the exclusive correlation between strength and ductility result from synergistic effects between thermodynamics and dislocation kinetics. The authors found that the plastic deformation of the BCC-Fe cell with the <100> and <112> orientations is dominated by strain twinning, while that of the <110> and <111> orientations is governed by dislocation slip. As a result of the conducted analyses, the authors indicated the low driving force (ΔG = 6.395 GPa) and high energy barrier (Q = 11.95 KJ/mol) of the BCC-Fe cell with <100> orientation as responsible for the low yield stress (σy = 10.15 GPa) and high ductility (ε = 0.5). In contrast, the high ΔG (17.37 GPa) and low Q (6.45 KJ/mol) of the BCC-Fe cell with <111> orientation correspond to the high σy (27.57 GPa) and low ε (0.21).

The study in [10] involves an analysis of 10 μm thick Ni foil-deposited electro-resistance. This analysis was conducted with suitability for industrial applications in mind using a thermomechanical analyzer (TMA) and glass transition temperature (Tg) differential scanning calorimetry (DSC). The TMA method allowed for the assessment of microthermal deformations and changes occurring in the microstructure and grains at different annealing temperatures. Based on microstructural observations, it was found that with an increasing temperature from 400 °C to 600 °C, grain growth occurred, which intensified after the application of stress, inducing superplastic deformation. For the tested foils, the maximum elongation at 400 °C was 306%, with an equiaxed grain structure retained after deformation at a rate of 1 × 10^−3^ s^−1^. Superplasticity was not observed at temperatures of 500 °C or 600 °C.

Ref. [11] presents a characterization of through-thickness shear anisotropy using the double-bridge shear test as well as finite element model updating based on shear strain measurements using the digital image correlation (DIC) method. This study was conducted on a 2.42 mm thick cold-rolled AW5754-H22 aluminum sheet. The use of the double bridge increased the sensitivity of the measurements to out-of-plane shear parameter disturbances. The study found that for the tested AW5754-H22 sheet, the force–strain responses measured in the y–z and x–z planes were 8% and 12% lower, respectively, compared to the responses in the x–y plane, among other findings. This result indicates the presence of through-thickness shear anisotropy.

Zhengming Wang, along with co-authors, use ab initio molecular dynamics simulations to directly calculate the diffusivities of O, Y, and Ti at four different temperatures in liquid 316 L stainless steel using a new on-the-fly machine learning force field function from the VASP software package version 6.3.2 [12]. The authors indicate the average diffusivities of the individual elements studied in liquid 316 L steel at temperatures of 1850, 2000, 2200, and 2500 K for the following, respectively:

- Oxygen: 2.89 × 10^−5^ cm^2^/s, 4.88 × 10^−5^ cm^2^/s, 7.74 × 10^−5^ cm^2^/s, and 1.79 × 10^−4^ cm^2^/s;
- Titanium: 1.90 × 10^−5^ cm^2^/s, 2.63 × 10^−5^ cm^2^/s, 4.11 × 10^−5^ cm^2^/s, and 7.14 × 10^−5^ cm^2^/s;
- Yttrium: 2.19 × 10^−5^ cm^2^/s, 2.77 × 10^−5^ cm^2^/s, 3.39 × 10^−5^ cm^2^/s, and 4.85 × 10^−5^ cm^2^/s.

The presented results provide important physical parameters that can be successfully used for future modeling of oxide deposition kinetics during additive manufacturing.

The microstructural changes in the TB6 (Ti-10V-2Fe-3Al) alloy composed of the α-phase and β-phase under the influence of planar wave detonation were the subject of investigation in [13]. By examining the deformation twin in grains at high strain rates, it was found that twin deformation occurs only in the α-phase. It was found that twin deformation operates only in the α-phase due to the limited slip system in this phase. The orientation of α changes from {1010} to {0002}, and the maximum intensity of the {1010} poles decreases due to the twinning process. The occurrence of twins of the <1210>/84.7° type was observed mainly in these grains. The deviation from the ideal twin misorientation was found to become large and cause a wider distribution of twin misorientations with increasing deformation. A twin with a different grain orientation will start to act differently. A certain selection of twin variants was also found, while the orientation of all {1012} twins was found to be oriented towards {0002} in different grains with different degrees of deformation.

The microstructural quality of two high-temperature aluminum alloys, A356 + 3.5RE and Al-8Ce-10Mg, was evaluated by comparison with T6 A356 [14]. These alloys were subjected to thermal conditioning at temperatures of 250 and 300 °C for 200 h. The Si phase was found to grow significantly (13–24%) in the T6 A356, A356 + 3.5RE, and T6 A356 + 3.5RE alloys. The growth of the silicon-rich phase resulted in a decrease in mechanical properties. Stabilization of the orthorhombic Al4Ce3Si6 phase and the monoclinic β-Al5FeSi phase was observed in the A356 3.5R alloy after T6 heat treatment. These phases increase the mechanical properties of the alloy but prevent its thermal conditioning. It was also found that T6 treatment reduced the volume of the cubic Al20CeTi2 phase by 13% and the hexagonal Π-Al9FeSi3Mg5 phase by 23%, which adversely affected the mechanical properties. After conditioning, an 82 to 101% increase in the volume of the cubic β-Al3Mg2 phase in the Al-8Ce-10Mg alloy was observed, leading to a decrease in the mechanical properties of the alloy.

Studies on the effect of non-metallic inclusions on the fatigue strength factor of high-purity steel containing an average of 0.23% C, 1.23% Mn, and 0.0025 B, industrially melted in 140-ton production furnaces, subjected to desulfurization and refined with argon, are presented in [15]. The steel was differentiated in terms of microstructure and mechanical properties by hardening and tempering at various temperatures. It was found that submicroscopic Al_2_O_3_ inclusions predominate in the tested steel. Large inclusions with diameters exceeding 5 um occur sporadically. Based on the analysis of the test results, it was found that fatigue resistance factor k decreases with increasing distances between impurities. A higher fatigue resistance factor was observed with a large proportion of smaller non-metallic inclusions. This may indicate that small, oval-shaped non-metallic inclusions do not reduce the fatigue life of steel, regardless of its microstructure. Furthermore, it was found that with small spacings between non-metallic inclusions, the tempering temperature, and therefore the microstructural and mechanical properties, has a stronger effect on the fatigue resistance factor, and this effect is higher than that with larger spacings between inclusions.

The literature review presented in [16] examines the history of Sn whiskers from their origin to the latest results. The authors focus on solutions to suppress their formation by modifying the composition of solder alloys. They highlight promising results achieved by adding Bi and In alloys. The paper emphasizes that adding TiO_2_ and ZnO nanoparticles to solder alloys can significantly increase the corrosion resistance of composite solder joints. This can lead to the inhibition of corrosion-induced whisker growth. However, SiC, CuO, and ZrO_2_ nanoparticles have been described to have opposite effects on the susceptibility of composite solder joints to whisker formation.

The results of research on microstructural changes in the corrosion resistance direction of metal alloys are also presented [17,18,19,20]. Ref. [17] presents the results of a study of UNS S 32750 steel casted and then heat-treated at 1100 °C, with air cooling. The effect of secondary-phase precipitation on the passivation layer was analyzed by analyzing the precipitation of secondary phases after heat treatment at 700 °C with quenching at 50 K/s. The authors demonstrated the formation of segregation of the alloying elements and a reduction in the passivation layer thickness from 25 μm to 20 μm. The authors noted that this thinning of the passive layer can reduce the durability of batteries. They also demonstrated the precipitation of a secondary phase in the UNS S 32750 alloy at 700 °C, starting at the austenite boundaries, among other findings. The chromium content in the ferrite was 26.6 wt.%, and that in the austenite was 23.3%. Due to the precipitation of undissolved Cr and Mo at the austenite boundaries, secondary-phase precipitation was observed, with a tendency to increase along the austenite boundaries. The secondary phase, Sigma, precipitated primarily due to Cr and Mo segregation, followed by the formation of the Chi phase due to Cr and Mo deficiencies. Additionally, N, which remains unincorporated into Sigma and Chi, precipitated as CrN. The precipitation of the secondary phase, influenced by Cr and Mo segregation, led to differences in the PREN. These differences in PREN contributed to galvanic corrosion and reduced corrosion resistance. Therefore, galvanic corrosion resulted from compositional changes. The corrosion resistance of DX57D steel with a double-sided hot-dip zinc coating (ZnAlMg) and cut using SECM and SVET techniques to simulate acid rain was analyzed in [18]. This paper reported that the cathodic activity at the cut edge developed heterogeneously. Local activation occurred at the sites of white corrosion product deposition. The alkaline pHs above the cathode favored the precipitation of zinc and magnesium hydroxides and carbonates. The high intensity of these processes was observed for ZM90 and ZM120. ZM70, with a density of 70 g/m^−2^, displayed an accumulation of Al and Mg in the form of binary and ternary eutectics, as well as oxides. Zinc was present mainly in metallic form. ZM90 and ZM120, with densities of 90 g/m^−2^ and 120 g/m^−2^, showed a higher concentration of zinc oxides and hydroxides, which favored the formation of white corrosion products. Mg and Al accumulated as eutectic phases at the iron–coating interface, especially in coatings with a density of 120 g/m^−2^. Analyzing the changes occurring in the alloy, the authors concluded that a thinner galvanized coating may provide better long-term corrosion resistance compared to thicker coatings, as the higher dissolution rate of the ZM70 coating may actually prevent the formation of visible and undesirable mechanical white rust, while also providing cathodic protection and more effectively preventing severe localized corrosion at the iron–coating interface. An analysis of the corrosion process of 304 stainless steel containing delta ferrite in a real wastewater environment, in a humid environment containing hydrogen sulfide, and in microorganisms found in wastewater in contact with the steel is presented in [19]. The authors confirmed the negative impact of delta ferrite, known from the literature to be a phase that causes the embrittlement of austenitic steel and, consequently, that promotes pitting corrosion, a very dangerous process for corrosion-resistant steels. This study confirmed the presence of bacteria involved in the reduction in sulfur or sulfate compounds, leading to the formation of hydrogen sulfide, responsible for the corrosion process of steel sheets operating in wastewater environments. The authors proposed an approach to corrosion processes that includes the identification of bacterial classes to assess wastewater aggressiveness and corrosive conditions. The surface segregation of dissolved substances and its effect on the anticorrosion properties of the Fe0.95Al0.05, Fe0.95V0.05, Fe0.90Al0.05V0.05, Fe0.95Ti0.05, and Fe0.95Ge0.05 alloys were analyzed in [20]. It was found that the Fe0.95Ti0.05 sample, after annealing under vacuum at 1073 K, had significantly better anticorrosion properties than the other alloys tested. The authors explained this effect as being due to the strong surface segregation of Ti, indicating this element to be the reason for the material’s good corrosion resistance. As a result of this analysis, the authors concluded that the corrosion resistance of the tested alloys was directly related to the surface concentration of the dissolved Al, V, and Ti compounds.

An analysis of the effect of cast iron chemical composition and its potential changes during the production cycle on the elastic properties and the correctness of the numerical solution of the natural vibrations of ventilated brake disks is presented in [21]. The authors noted that increasing the degree of saturation of eutectic cast iron with flake graphite from 0.87 to 1.01 resulted in a 21% decrease in Young’s modulus and a decrease in the first natural frequency of the brake disks by approximately 10%. They also found that even small changes in the cast iron’s chemical composition can affect the frequency stability of the disks, while the high sensitivity of the first natural frequency to changes in the degree of the eutectic saturation coefficient, SC, requires the use of high-quality input materials, as well as strict control of the cast iron production process, at the melting and modification stages.

The results of a quantitative analysis of the mechanism of the influence of welding current on weld formation are presented in [22]. In this paper, a three-dimensional magnetohydrodynamic arc model with a variable load of electrode-negative EN and electrode-positive EP arcs is presented to quantitatively analyze the arc characteristics. The authors found that the EN phase arc is characterized by a higher penetration force and a keyhole effect, while in the EP phase, arc divergence occurs. It was shown that the heat and arc force are subject to periodic fluctuations related to polarity. The fluctuation in the long-axis hole size and hole area corresponding to the “critical current difference” is minimal, with the long-axis hole size ranging between 4.5 and 5.2 mm and the hole area ranging between 78 and 83 mm^2^.
